# It's Not That Great Anymore: The Central Role of Defense Mechanisms in Grandiose and Vulnerable Narcissism

**DOI:** 10.3389/fpsyt.2021.661948

**Published:** 2021-06-11

**Authors:** Leonie Kampe, Johannes Bohn, Carina Remmers, Susanne Hörz-Sagstetter

**Affiliations:** ^1^Department of Clinical Psychology and Psychotherapy, Psychologische Hochschule Berlin, Berlin, Germany; ^2^Department of Psychiatry, Psychotherapy and Psychosomatic Medicine, Psychosoziales Zentrum Itzehoe, Itzehoe, Germany; ^3^Department of Education and Psychology, Freie Universität Berlin, Berlin, Germany; ^4^Faculty of Health/School of Psychology and Psychiatry, Witten/Herdecke University, Witten, Germany

**Keywords:** grandiose narcissism, vulnerable narcissism, defense mechanisms, psychological distress, personality, emotion regulation, adaptive functioning, dimensional assessment

## Abstract

**Objectives:** The concept of narcissism contains a yet unresolved paradox: Its grandiose facet depicts the psychopathological core but is often associated with life-satisfaction and overall functioning, whereas its vulnerable facet is associated with psychological distress, but still not included in the international classification systems. Our goal was to investigate the relationship between the two facets of narcissism expecting underlying defense mechanisms to be core elements. First, we aimed to identify defense mechanisms specific to grandiose and vulnerable narcissism. Second, we explored how both facets are differentially associated with psychological distress, assuming that grandiose narcissism would be associated with less psychological distress than vulnerable narcissism. Third, we investigated the mediating role of defense mechanisms between narcissism and psychological distress.

**Methods:** In a non-clinical sample of *N* = 254 individuals, the Pathological Narcissism Inventory was used for the assessment of grandiose and vulnerable facets of narcissism, the Defense Style Questionnaire for defense mechanisms, and the Brief Symptom Inventory for psychological distress. Structural equation modeling was employed to identify distinct factors of grandiose and vulnerable narcissism. Associations between specific defense mechanisms and both facets were calculated. Furthermore, the direct association between both facets and psychological distress was examined. We finally explored whether defense mechanisms mediate the association between distress and both grandiose and vulnerable narcissism.

**Results:** A distinct pattern of defense mechanisms for each facet of narcissism could be extracted: Both facets showed significant positive correlations with specific intermediate and all maladaptive defense mechanisms. Only grandiose narcissism showed significant positive correlations with adaptive defenses. Vulnerable narcissism showed negative correlations with all adaptive defenses. Specifically, grandiose narcissism was significantly related to anticipation, pseudo-altruism, rationalization, and dissociation, whereas vulnerable narcissism was negatively related to all these defense mechanisms. While grandiose narcissism was not related to psychological distress, vulnerable narcissism showed high correlations with psychological distress. Intriguingly, mediator analysis found that grandiose narcissism was related to psychological distress when mediated by maladaptive defense mechanisms.

**Discussion:** The role of defense mechanisms is central for a differentiated understanding of the two different faces of narcissism. The relevance of assessing defense mechanisms in clinical settings, and related empirical findings are discussed.

## Introduction

The concept of narcissism plays a central role in personality research as well as in clinical psychological practice. Due to the seemingly contradicting manifestations of narcissism, understanding the underlying mechanisms is of both theoretical and practical importance. On the one hand, narcissism, as a personality trait, is related to numerous positive factors such as socio-economic success, overall life satisfaction, as well as psychological health (1, 2). On the other hand, high expressions on the continuum of narcissism are associated with proneness to emotional crisis, attachment anxieties, problematic long-term relationships, and severe problems in psychotherapy such as emotional reticence, unwillingness to change, and a higher drop-out rate (3–6).

Especially in clinical diagnostic settings, narcissistic pathology is often overlooked, and treatments are classified as seemingly “going well.” Eventually, when confronted with unexpected dropout, crises about upcoming separations from the therapist, or when patients change only little over the course of the treatment (7, 8), the underlying vulnerability and dysfunctionality of narcissism becomes evident. Many controversies surrounding the concept of narcissism and its clinical manifestations may be rooted in its one-sided operationalization in the international classification systems (9–11). The current definition of pathological narcissism in DSM-5 predominantly relates to the grandiose manifestation, consisting of a sense of entitlement, an excessive need for admiration, arrogant and self-centered behaviors, a proneness to envy and devaluation of others, and a lack of empathy and exploitative behaviors (12). Emerging consensus criticizes this definition by calling out its conceptual narrowness. Specifically, the definition of narcissism in DSM-5 neglects a different, more vulnerable side of this phenomenon (9, 13, 14). Psychoanalytic theory, empirical evidence, and clinical manifestations point to another facet of narcissism that captures specific insecurities underlying grandiose manifestations (15, 16). Following this theory, grandiose narcissism is understood as a defensive shield that is rigidly and unconsciously built up to defend the conscious ego from threats to the self-esteem (17). This theoretical conceptualization helps to understand why grandiose narcissism operates as a defensive structure that is related to indicators of psychological health, whereas its underlying vulnerability is not. By calling it a character defense, the defensive structure of grandiose narcissism may itself be seen as the core of the narcissistic pathology. Following this line of thought, it becomes essential to address defense mechanisms in psychotherapeutic treatment in order to access underlying vulnerabilities and their related psychological problems (17).

In spite of its clinical vividness, this complex psychoanalytic relationship has not yet been fully investigated empirically. To fill this gap, the current study has the goal to investigate the quality and functional role of defense mechanisms in grandiose and vulnerable manifestations of narcissism and their associations with the experience of psychological distress.

### Defense Mechanisms

The idea that specific manifestations of narcissism are related to a distinctive defensive structure has been thoroughly elaborated in psychoanalytic literature (17, 18). Defense mechanisms are conceptualized as unconscious mental operations that regulate internal and external conflicts implicitly (19, 111). Defense mechanisms that are assumed to play a central role in narcissism are related to severe anxieties (20) and shame (21). With regard to their functionality, defense mechanisms can be clustered hierarchically and spanned over a continuum ranging from adaptive, over intermediate (neurotic), to maladaptive (pathological) mechanisms (22). Adaptive defense mechanisms such as humor, anticipation, and suppression help the individual to deal with unpleasant emotional experiences such as ambivalences or distressing realities. They can be used flexibly and reduce negative affective responses successfully. For example, in a situation in which a person embarrasses herself, she may circumvent the aversive feeling of being ashamed by making a joke. Adaptive defenses are related to psychological health and negatively associated with personality pathology (22). Intermediate (or in psychoanalytic terms: neurotic) defenses are also unconsciously applied to regulate emotional distress. Unlike adaptive defenses, they are used more rigidly and aim to avoid the experience of upsetting emotions. One of the functions of intermediate/neurotic defense mechanisms can be seen to keeping aggression away from important relationships. For example, a person who feels attacked by a colleague may hug her effusively at the next encounter and hereby transform the initial anger into its opposite, an unconscious mental transformation also called reaction formation. Examples for intermediate/neurotic defenses are turning against the self, pseudo-altruism or reaction formation. They can be helpful when applied with flexibility but are moderately related to the internal experience of psychological distress (22).

Maladaptive defenses, on the other end of the spectrum, are mechanisms to exclude potentially threatening emotional negative affects from the self by, for example, projecting them on other people or by dissociating from them. Examples are projection, splitting, and projective identification. In contrast to more adaptive defenses, that operate on an intrapsychic level, maladaptive defenses are employed mainly interpersonally, hence using others to (unconsciously) regulate one's own emotional distress. The dominant use of these defense mechanisms is strongly related to relationship problems, psychiatric disorders, and personality pathology (23–28).

### Narcissism and Defenses

From an etiologic point of view, defense mechanisms in narcissism are understood as a developmental consequence from early experiences of rejection and devaluation by primary caregivers (29, 30). In this context, it is argued that the grandiose manifestation of narcissism result from an unconscious compensatory process to defend oneself against severe anxieties, shame, and threats to the self-esteem (18, 20, 21). By coining the term “character defense”, Kernberg (17) argues that the core of the narcissistic pathology can be seen in a defensive operation to sustain the ego by splitting based, projective and reality-exceeding defensive operations such as grandiose fantasies, omnipotence, devaluation and idealization of the self and others, denial and externalization. To date, there are only few empirical studies that have investigated mechanisms that are specifically related to narcissism: Perry and Perry (31) found devaluation, omnipotence, idealization, and mood-incongruent denial as specific narcissistic defensive operations. Hilsenroth et al. (32) found idealization, and Raskin and Novacek (33) identified grandiose fantasies as defense mechanisms as specifically related to narcissism. The unconscious use of these mechanisms has the goal of preventing unpleasant realities from the consciousness to sustain the world of omnipotence, importance, and grandiose fantasy (17).

### Grandiose and Vulnerable Narcissism

The concept of narcissism has widely been formed by the grandiose phenotype which, to date, still is the underlying concept in the international classification systems of psychiatric diseases (34, 35). Due to criterion problems and related inconsistencies (36), the mere focus on the grandiose side of narcissism was criticized and subsequently investigated and revised (9). In spite of former differentiations between normal and pathological narcissism, there is a growing consensus toward a dimensional conceptualization with normal and pathological narcissisms as two poles of the spectrum (14). Furthermore, research on underlying factors of narcissism has emerged: Numerous studies found different factor structures in narcissism: Besides five (37), and three (16, 38, 39) factor solutions, prevailing evidence supports the assumption of two distinct factors in narcissism, namely grandiose (GN) and vulnerable (VN) narcissism (9, 16, 40–45).

While psychoanalytic theory suggests VN to be the underlying insecurity of GN, empirical research suggests that both are distinct factors of narcissism with fluctuating expressions (41, 46–48). Intriguingly, the two facets of narcissism show very distinct clinical appearances. GN is linked to higher self-esteem, self-construal, and extraversion (2, 41, 49) and associated with higher sensibility to achievement setbacks (50). It is furthermore related to a hedonistic orientation and risk-taking behavior, impulsivity, and little consideration for future consequences (48) and also related to less treatment utilization and more drop-out (4). VN on the contrary is related to lower self-esteem, interdependent self-construal, attachment anxiety (49), introversion (41), sensitivity to shaming interpersonal experiences (50), a fatalistic and negative life perspective (48), and a hostile attribution bias (112).

While GN is generally associated with better psycho-social functioning, life satisfaction, and psychological health (1, 2), VN is related to neuroticism (51), higher psychological distress and depressive symptoms (1, 52), and less life satisfaction (2). It is also associated with difficulties in accessing adaptive emotion regulation strategies (53) and overall considered to be more dysfunctional. While GN is related to narcissistic personality disorder, VN is related to borderline personality disorder with severe impairments in psychological functioning (54).

Due to the clinical relevance and the particular relationship between narcissistic features and clinical challenges, treatment difficulties and lack of therapeutic response (3, 6, 7), the concept of narcissism has gained increasing attention in clinical conceptualizations and empirical research. The role of emotion regulation strategies related to narcissism has thereby shown to be of central clinical relevance. Recent studies have examined the relationship between dimensions of pathological narcissism and depressive symptoms, finding a consistent association between pathological narcissism and depressive symptoms in a longitudinal design (55), discussing emotional processing abilities as possible mediator (in VN) (56), and the role of dysfunctional attitudes like perfectionism in explaining the link between VN and depression (57).

Another avenue of research is the finding of a robust and projective defensive structure as a central factor in complications, refusals of change, drop-outs or stagnating treatment courses (3, 7, 8, 20, 58, 59). A study with narcissistic psychiatric outpatients showed an association between high levels of narcissism and greater interpersonal impairment by engaging in domineering, vindictive, and intrusive behaviors and a failure to complete treatment (5, 60). Mielimaka et al. (61) later found that the defensive style mediated the relationship between narcissism and interpersonal problems: Albeit narcissism was not directly related to interpersonal problems, they found an indirect effect when mediated by neurotic defense mechanisms. Ultimately, the differentiation between GN and VN has shown to be of informative value: Studies on the relationship between pathological narcissism (GN and VN), defensive functioning, and coping abilities have shown that GN and VN are associated with diverging coping strategies (62). VN, but not GN, is associated with hostile attribution bias, which could be interpreted as projective processes (63), and VN was strongly associated with narcissistic rage, hostility and aggressive behavior (64). The diverging relationships of GN and VN with emotion regulation strategies hence seem to be of high clinical relevance and deserve further investigation.

### Aims of the Current Study

Our aim was to further elucidate this issue by considering the role that defense mechanisms play in the paradoxical relationship between narcissism and psychological distress. Firstly, we aimed to explore specific defense mechanisms that are used in GN and in VN, respectively. Secondly, we aimed to explore the differential associations between GN and VN and psychological distress. Thirdly, we assumed that taking defense mechanisms into consideration might shed light on the relationship between narcissism and distress and may thus help to resolve the contradictions between grandiose narcissism and its ambiguous association with psychological distress. For this we conducted a cross-sectional study in which we assessed GN, VN, defense mechanisms and indicators of psychological distress in a non-clinical sample.

## Materials and Methods

Our study has been preregistered at Open Science Forum (OSF). A detailed description of the research project and the full study plan can be accessed via the following link https://osf.io/9tuqd/.

### Participants

A non-clinical sample of *N* = 254 (192 females, 59 males, and three with no specified gender) individuals was recruited via university and general mailing lists and assessed by an online survey as part of a larger study on personality, defenses and attachment (not relevant for the current thrust). Approval of the ethics committee of the Psychologische Hochschule Berlin was obtained. Inclusion criteria was a minimum age of 18 years and sufficient German language skills. A descriptive analysis of the sample is given in Table 1.

**Table 1:** Descriptive statistics.

	**Mean**	**SD**	**Min**	**Max**
Age	33.56	15.03	18	73
Psychological distress	1.73	0.63	1.00	4.28
Grandiose narcissism	3.19	0.75	1.40	5.15
Vulnerable narcissism	3.04	0.79	1.00	5.56
**Adaptive defense mechanisms**				
Suppression	4.21	1.75	1	9
Anticipation	5.36	1.69	1	9
Humor	5.52	1.76	1	9
Sublimation^*^	3.70	2.23	1	9
Rationalization^*^	6.23	1.58	2	9
Denial^*^	2.46	1.73	1	9
Dissociation	3.10	1.58	1	7.5
**Intermediate/neurotic defense mechanisms**				
Pseudo-altruism	4.96	1.44	1	8.5
Undoing	4.08	1.75	1	9
Reaction formation^*^	4.45	2.21	1	9
Acting out	3.53	1.75	1	8.5
**Maladaptive defense mechanisms**				
Splitting	2.68	1.64	1	7.5
Autistic fantasy	3.11	2.06	1	9
Projection	2.17	1.42	1	8
Passive aggression	2.50	1.52	1	9
Idealization	3.10	1.58	1	7.5
Somatization	3.35	1.84	1	9
Isolation	3.25	1.94	1	9
Displacement	3.48	1.84	1	9
Devaluation	3.28	1.54	1	8.5

*For all scales, the mean values over all items are displayed. For psychological distress the rating scales ranged from 1 to 5; for grandiose and vulnerable narcissism the ratings scales ranged from 1 to 6; for the defense mechanisms the rating scales ranged from 1 to 9*.

* *For this defense mechanism only one item was used*.

### Measures

#### Narcissism

For the assessment of grandiose and vulnerable narcissism, we used the German version of the Pathological Narcissism Inventory [PNI, (65); English original version: (44)]. The German PNI is a multidimensional measure for grandiose and vulnerable features of pathological narcissism and contains 54 items. It includes a translation of the 52 items of the original English PNI plus two additional items for the exploitative subscale, constructed and validated by the authors of the German version. The PNI consists of the following seven subscales: exploitativeness (EXP, seven items, e.g., item 15: “I find it easy to manipulate people”), grandiose fantasy (GF, seven items, e.g., item 42: “I often fantasize about performing heroic deeds”), self-sacrificing self-enhancement (SSSE, six items, e.g., item 22: “I feel important when others rely on me”), entitlement rage (ER, eight items, e.g., item 29: “I get angry when criticized”), devaluing (DEV, seven items, e.g., item 17: “Sometimes I avoid people because I'm concerned that they'll disappoint me”), contingent self-esteem (CSE, 12 items, e.g., item 36: “It's hard to feel good about myself unless I know other people like me”), and hiding the self (HS, seven items, e.g., item 9: “I often hide my needs for fear that others will see me as needy and dependent”). Items are scored on a 6-point Likert scale ranging from 0 (*not at all like me*) to 5 (*very much like me*). The PNI shows overall good psychometric properties with alpha coefficients ranging between α = 0.82 and α = 0.92. Re-test reliability for the total score was at α = 0.86 and CFA and ESEM confirmed the 7-factor lower order factor structure (65). Conclusions for higher order factor structures still remain open, however, empirical evidence suggests a two-factor solution for grandiose narcissism consisting of factors EXP, GF, and SSSE, and vulnerable narcissism consisting of factors ER, DEV, HS and CSE (44, 65–68). We based our analyses on this two-factor solution.

#### Defense Mechanisms

The Defense Style Questionnaire [DSQ 40, (69)] is the 40 item German version of the English DSQ 40 (70). In the DSQ, 20 defense mechanisms, represented by two items each, are assessed on a 9-point Likert scale ranging from 1 (*strongly disagree*) to 9 (*strongly agree*). The items can be classified into three categories, each forming an individual scale: adaptive, intermediate (neurotic), and maladaptive defense mechanisms. The respective items are marked as such in the results. Factor analysis of the German version confirmed the original factor structure but assigned individual defense mechanisms to the three factors in a different pattern: adaptive defenses (sublimation, humor, anticipation, suppression, rationalization, dissociation, and denial), intermediate/neurotic defenses (pseudo-altruism, undoing, reaction formation, and acting out), and maladaptive defenses (splitting, autistic fantasies, projection, passive aggression, idealization, somatization, isolation, displacement, and devaluation) (69). Other studies on the DSQ 40 vary in the assignment of the individual defense mechanisms to their levels of adaptiveness. For our study we based the assignment on the factor analysis of Schauenburg et al. (69), although some clincal doubts might remain. However, since we did not use the scale means for our analysis, the assignment is irrelevant for the interpretation of our results.

#### Psychological Distress

For the assessment of psychological distress, we used the German version of the Brief Symptom Inventory [BSI-18; German version: Mini-SCL, (71)]. The BSI-18 is a reliable and short instrument for the assessment of clinical distress to assess subjective mental impairment on the scales depression (α = 0.87), anxiety (α = 0.84), and somatization (α = 0.82) (72). Items are rated on a 5-point Likert scales ranging from 0 (*not at all*) to 4 (*extremely*). For this study, we used the Global Severity Index (GSI, α = 0.93), an overall score for psychological distress that can be calculated from the three subscales. Both, first and second order factor structures were supported by CFA (72).

### Statistical Analysis

For an a priori calculation of the sample size, comparable studies served as orientiation [e.g., (73, 74)] for the calculation of the correlations. These studies show an average effect sizes of *r* = 0.3. A power analysis for the calculation of the sample size was conducted with the program G^*^Power 3.1 (75). With an alpha error probability of 0.05, an estimated power of 0.95 and the estimated effect size of *r* = 0.3, a sample size of 134 participants resulted from the analysis. As structural equation models require a minimum of *N* = 200 (76) we used this as the study's benchmark.

**Figure 1:**
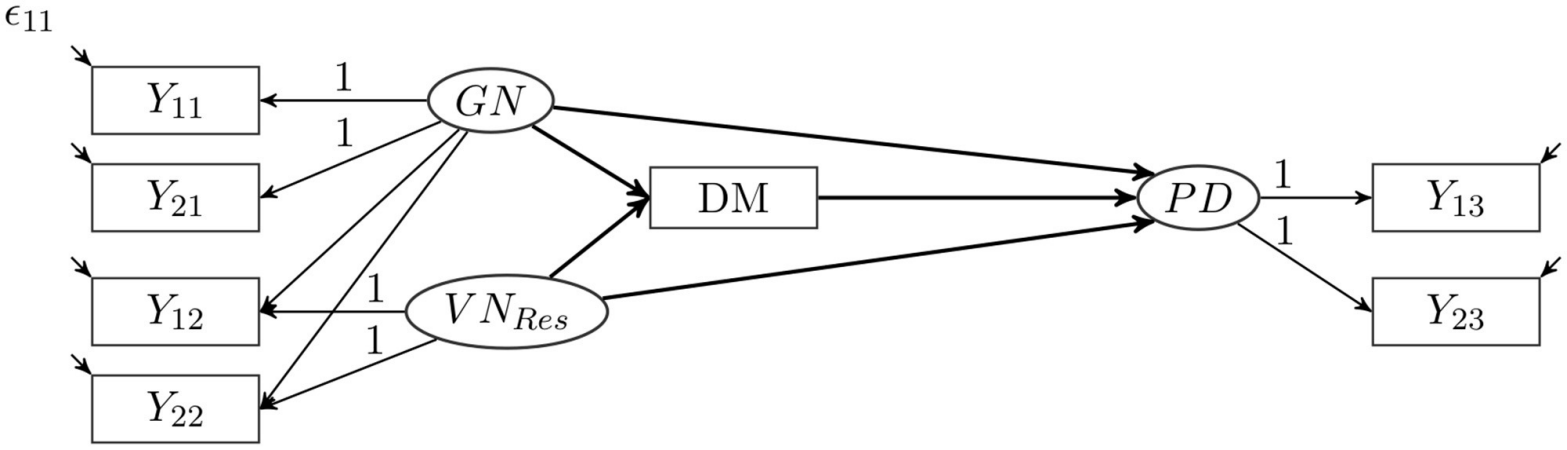
Structural equation model of grandiose and vulnerable narcissism, defense mechanisms, and psychological distress.

Structural equation modeling was used to address the questions in this study. These models combine the different facets of narcissism (assessed by PNI), defense mechanisms (assessed by DSQ 40), and psychological distress (assessed by BSI-18) into one model. The model is displayed in Figure 1.

To represent the different correlations of GN and VN with the other variables, we used a bifactor S-1 model (77, 78). This S-1 model allows a clear separation of grandiose and vulnerable aspects of narcissism. Since GN is the core of the current definition of pathological narcissism, it was chosen as the reference factor for the model. Based on prior research and modeling suggestions (44, 65, 68) two parcels (*Y*_11_ and *Y*_21_) were calculated from the items of the PNI scales for EXP, GF, and SSSE. These two parcels load on the GN factor, which represents the degree of grandiose narcissism. Two other parcels (*Y*_12_ and *Y*_22_) were calculated from the remaining items of the PNI, which form the CSE, DEV, ER, and HS scales. These two parcels also load on the GN factor. These two parcels additionally load on a second factor VN_Res_ that is uncorrelated with the GN factor. This second factor is a residual factor, and it describes that portion in the variance of vulnerable narcissism that cannot be explained by GN. This residual factor has the mean value 0 and a person with a value of 0 in this factor would have exactly the value in the VN that would be expected on the basis of the GN. Thus, a person with a positive value on this residual factor would have a higher VN than one would have expected based on their GN. Through this approach, the GN factor represents the grandiose elements of narcissism, encompassing the elements contained in both GN and VN. The residual factor of VN contains only those elements that have nothing in common with GN. By separating the reference and the residual factor, we can better examine the influence on other variables the specific vulnerable facet of narcissism has independently of grandiose narcissism.

For each defense mechanism, an individual model was calculated, leading to a total of 20 models. Each defense mechanism also formed a mediator between the two narcissism factors and psychological distress. Two parcels (*Y*_13_ and *Y*_23_) were calculated from the items of the BSI-18 and loaded on the psychological distress factor. We used this model to examine the strength of the relationship between the narcissism factors and the respective defense mechanism, the strength of the influence on psychological distress, and to what extent this influence is mediated by the defense mechanism.

The defense mechanisms were each comprised of two items and the mean of these two items was used as a manifest variable in the model. For the defense mechanisms of sublimation, rationalization, reaction formation, and denial, the correlations of the two items were not significant. In each of these models, only the item that represented the defense mechanism best in terms of content was used.

The model was evaluated with Mplus 8 using a maximum likelihood estimator. The goodness-of-fit of all models was examined with the χ^2^-Test, the CFI, and the RMSEA. A good model fit is indicated by a value of χ^2^ < 2^*^df, a CFI > 0.97, and a RMSEA < 0.05; an acceptable model fit is indicated by a value if χ^2^ < 3^*^df, a CFI > 0.95, and a RMSEA < 0.08 (79).

## Results

The model fit of all models are displayed in the Appendix. In 14 models the model fit was good and in five models the model fit was acceptable (in these models, the RMSEA was above 0.05 but below 0.08; the other model fit indices indicated a good model fit). Only in the model with projection, the model fit was too low (RMSEA = 0.092 and χ^2^ = 34.48 with df = 11). The results of this model should be interpreted with caution. The results of this study use the standardized regression coefficients *b* of the structural equation model, the size of which can be interpreted as correlations.

Descriptive statistics are displayed in Table 1. As expected in a non-clinical sample, participants used more adaptive defense mechanisms such as rationalization, humor, and anticipation than other defense mechanisms. Suppression and intermediate/neurotic defense mechanisms were employed occasionally, and maladaptive defense mechanisms were reported least frequently. In terms of psychological distress, we found rather low levels (*M* = 1.73, *SD* = 0.63) which is also expected in a non-clinical sample. Similar scores for GN (*M* = 3.19, *SD* = 0.75) and VN (*M* = 3.04, *SD* = 0.79) were found. The associations between the specific defense mechanisms and psychological distress are depicted in Table 2.

**Table 2:** Direct and indirect effects of narcissism on psychological distress mediated by defense mechanisms.

**Model**	**Effects on DM**		**Effects on psychological distress**				
	**GN**	**VN_**Res**_**	**DM**	**GN direct**	**GN indirect**	**VN_**Res**_ direct**	**VN_**Res**_ indirect**
**Adaptive**							
Suppression	0.070	−0.265^*^	−0.028	0.110	−0.002	0.492^*^	0.007
Anticipation	0.263^*^	−0.009	−0.012	0.112	−0.003	0.499^*^	0.000
Humor	0.010	−0.194^*^	0.078	0.105	0.001	0.514^*^	−0.015
Sublimation	0.071	0.000	0.126^*^	0.101	0.009	0.501^*^	0.000
Rationalization	0.129^*^	−0.286^*^	−0.046	0.114	−0.006	0.486^*^	0.013
Denial	0.109	0.100	0.006	0.107	0.001	0.499^*^	0.001
Dissociation	0.219^*^	−0.187^*^	0.034	0.101	0.007	0.505^*^	−0.006
**Intermediate/neurotic**							
Pseudo-altruism	0.335^*^	−0.015	−0.018	0.113	−0.006	0.499^*^	0.000
Undoing	0.344^*^	0.364^*^	0.126	0.062	0.043	0.453^*^	0.046
Reaction formation	0.313^*^	0.308^*^	0.187^*^	0.047	0.058^*^	0.441^*^	0.058^*^
Acting out	0.306^*^	0.152^*^	0.236^*^	0.032	0.072^*^	0.464^*^	0.036^*^
**Maladaptive**							
Splitting	0.411^*^	0.245^*^	0.240^*^	0.011	0.099^*^	0.443^*^	0.059^*^
Autistic fantasy	0.323^*^	0.348^*^	0.259^*^	0.025	0.084^*^	0.410^*^	0.090^*^
Projection	0.232^*^	0.424^*^	0.235^*^	0.056	0.054^*^	0.400^*^	0.100^*^
Passive aggression	0.279^*^	0.338^*^	0.211^*^	0.051	0.059^*^	0.429^*^	0.071^*^
Idealization	0.355^*^	0.189^*^	0.157^*^	0.050	0.056^*^	0.471^*^	0.030^*^
Somatization	0.262^*^	0.370^*^	0.380^*^	0.009	0.100^*^	0.360^*^	0.141^*^
Isolation	0.167^*^	0.210^*^	0.247^*^	0.069	0.041^*^	0.450^*^	0.052^*^
Displacement	0.299^*^	0.435^*^	0.258^*^	0.030	0.077^*^	0.388^*^	0.112^*^
Devaluation	0.226^*^	0.278^*^	0.166^*^	0.071	0.037^*^	0.455^*^	0.046^*^

*The 20 models differ in the defense mechanism*.

*DM = defense mechanism; GN = grandiose narcissism factor; VN_Res_ = vulnerable narcissism residual factor*.

* *Significant effect*.

### Associations Between Grandiose and Vulnerable Narcissism and Specific Defense Mechanisms

**Figure 2:**
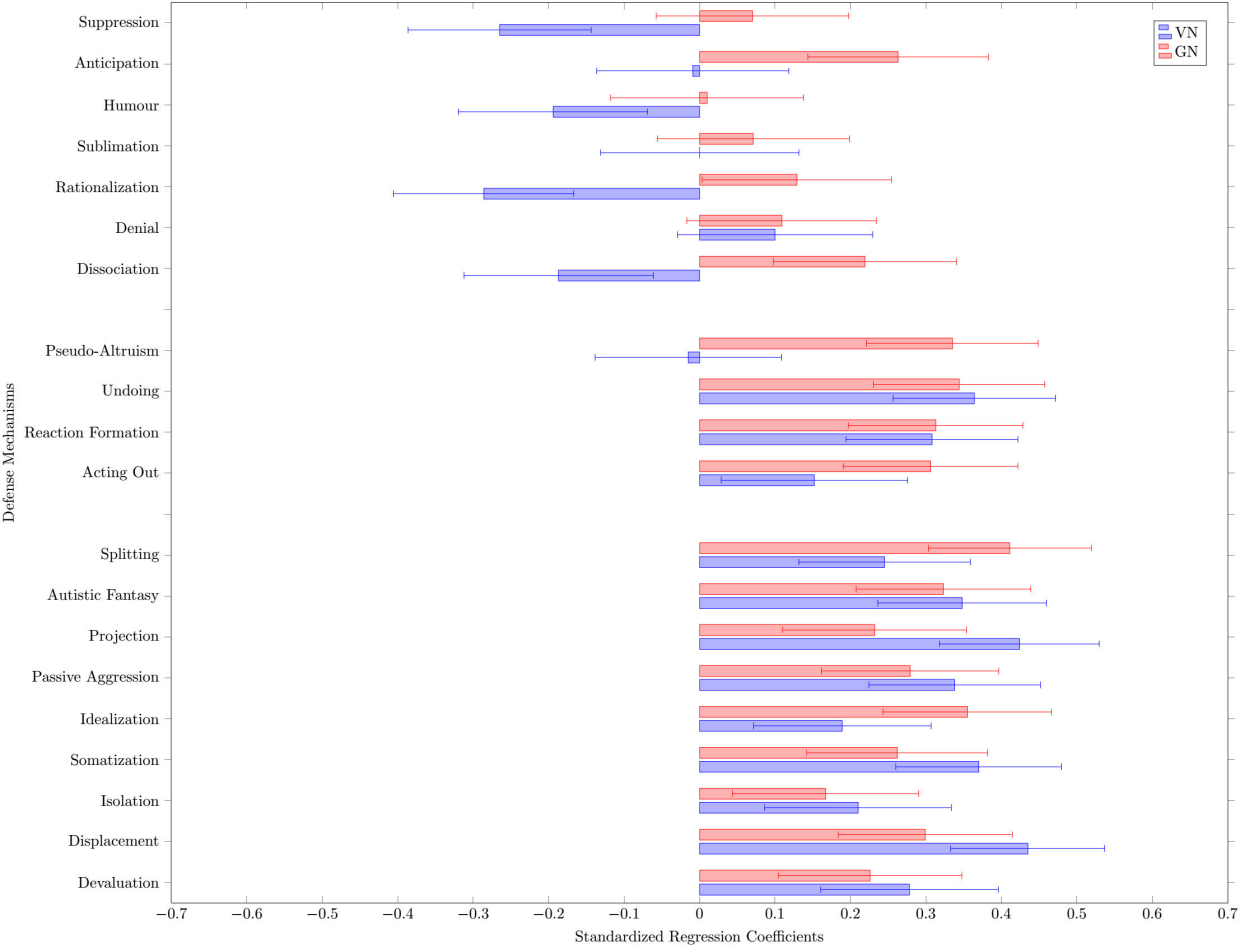
Influence of grandiose and vulnerable narcissism on defense mechanisms: standardized regression coefficients and their confidence intervals.

The associations are displayed in Figure 2 and can also be found in Table 2. GN showed significant positive associations with adaptive defense mechanisms anticipation (*b* = 0.26), rationalization (*b* = 0.13), and dissociation (*b* = 0.22); intermediate/neurotic defense mechanisms pseudo altruism (*b* = 0.36), undoing (*b* = 0.34), reaction formation (*b* = 0.31), and acting out (*b* = 0.31); and maladaptive defenses splitting (*b* = 0.41), idealization (*b* = 0.36), autistic fantasies (*b* = 0.32), displacement (*b* = 0.30), passive aggression (*b* = 0.28), somatization (*b* = 0.26), devaluation (*b* = 0.23), projection (*b* = 0.23), and isolation (*b* = 0.17).

VN showed significant negative associations with the adaptive defense mechanisms rationalization (*b* = −0.29), suppression (*b* = −0.27), humor (*b* = −0.19) and dissociation (*b* = −0.19). VN showed significant positive associations with the intermediate/neurotic defenses undoing (*b* = 0.36), reaction formation (*b* = 0.31), and acting out (*b* = 0.15) and with the maladaptive defenses displacement (*b* = 0.44), projection (*b* = 0.42), somatization (*b* = 0.37), autistic fantasies (*b* = 0.35), passive aggression (*b* = 0.34), devaluation (*b* = 0.28), splitting (*b* = 0.25), isolation (*b* = 0.21), idealization (*b* = 0.19).

Overall, in this non-clinical sample, GN seemed to be associated with most adaptive defense mechanisms and especially with all maladaptive and intermediate/neurotic defense mechanisms, while VN was negatively associated with adaptive mechanisms and strongly positively associated with maladaptive and neurotic defense mechanisms.

### Associations Between Grandiose and Vulnerable Narcissism and Psychological Distress

In order to estimate the association of narcissism and psychological distress regardless of a defense mechanism, a reduced model was estimated. This model is like the model in Figure 1, but without the defense mechanism. This reduced model had a very good model fit (χ^2^ = 9.89, *df* = 8, *p* = 0.27, RMSEA = 0.031, CFI = 0.99). In the reduced model, GN had no significant association with psychological distress [*b* = 0.107, *p* = 0.108, 95%-KI: (−0.024; 0.238)]. The residual factor of VN had a significant, positive association with psychological distress [*b* = 0.500, *p* < 0.001, 95%-KI: (0.396; 0.603)]. This is a large association. Participants who reported higher vulnerable narcissism than expected based on their grandiose narcissism reported more psychological distress.

### Mediator Analysis of Defense Mechanisms Between Narcissism and Psychological Distress

The results for the mediation analysis can be found in the last four columns of Table 2. Both GN and VN had indirect effects on psychological distress. The indirect effects were mediated by the corresponding defense mechanism of the model. No significant direct effect of GN on psychological distress was found. In contrast, strong direct effects of VN on psychological distress were found. GN showed significant indirect effects on psychological distress when mediated by specific defense mechanisms. More specifically, this mediation was found in models with reaction formation and acting out (from the intermediate/neurotic defense category) and in all models with maladaptive defense mechanisms. For VN, significant indirect effects on psychological distress mediated by the defense mechanisms could be found for the same models. This means, that individuals with higher levels of vulnerable and grandiose narcissism reported more maladaptive defense mechanisms and therefore more psychological distress.

## Discussion

The aim of the current study was to investigate the nature and role of defense mechanisms in grandiose and vulnerable facets of narcissism in a non-clinical sample. First, we aimed to explore defense mechanisms that are typical for GN and VN, respectively. Second, we aimed to replicate past findings showing that GN and VN are differentially related to psychological distress, assuming that GN would not be related to psychological distress and VN would be strongly related to psychological distress. Third, we assumed that specific defense mechanisms would shed light on the former differential narcissism-distress interplay and therefore explored whether and which defense mechanisms mediated the association between psychological distress and VN and GN, respectively.

To address our research questions we analyzed data of *N* = 254 healthy subjects with structural equation modeling and employed a bifactor S-1 model (77, 78). The latter allowed us to separate statistically grandiose and vulnerable aspects of narcissism. Since GN is the core of the current definition of pathological narcissism, it was chosen as the reference factor for the model.

Overall, specific defense mechanisms for both types of narcissism could be found. First, we found differences between GN and VN and the adaptiveness of their defensive structure: Both GN and VN were related to almost all intermediate/neurotic and maladaptive defense mechanisms. However, only GN was significantly positively related to the use of adaptive defense mechanisms. Since the use of adaptive defense mechanisms is related to mental health, this finding might be one of the explanations why GN is not associated with psychological distress while VN is. Second, we found that those defense mechanisms that were exclusively found in GN, were not only non-related, but significantly negatively related to VN. These mechanisms are pseudo-altruism, rationalization, anticipation, and dissociation. Only these mechanisms did not mediate the relationship between GN and psychological distress. This leads to the assumption that the use of these particular mechanisms in GN might be the strategical “advantage” of GN compared to VN when regulating psychological distress. Third, we found overall qualitative differences with regard to the defense mechanisms for GN and VN: The defense mechanisms showing the strongest association with grandiose narcissism are splitting-based (e.g., splitting, idealization, and devaluation) and socially desirable (e.g., pseudo altruism, anticipation, and rationalization). VN on the contrary was most strongly associated with defense mechanisms that can be summarized as related to dissociating the affect from the self (e.g., somatization, projection, autistic fantasies, and displacement) and self-directed defense mechanisms (e.g., reaction formation, undoing, and passive aggression). Overall, GN appeared to be related to the more effective and more socially desirable defensive styles than VN.

The current findings are in line with existing research on defense mechanisms in narcissism: Zeigler-Hill and Besser (80) found that GN was positively related with the use of adaptive humor (self-enhancing and affiliative), whereas VN was negatively associated with adaptive humor and positively with maladaptive humor (self-defeating and aggressive). Richardson and Boag (81) found defense mechanisms acting out, dissociation, and splitting for grandiose psychopathological narcissism and further showed that immature defensive strategies mediate the relationship between Machiavellianism and distress. Fernie et al. (62) found denial to be especially prominent in VN. Mielimaka et al. (61) reported a strong relationship between immature and neurotic defenses based on the DSQ and pathological narcissism, albeit not differentiating between GN and VN. To our best knowledge, the only existing study on defense mechanisms, differentiating between GN and VN, has recently been published by Khodabakhsh Pirklany and Safaeian (82), finding high expressions of GN and VN related to intermediate/neurotic and maladaptive defenses, and this being significantly higher than for individuals with low expressions in pathological narcissism.

With regard to psychological distress, GN was not directly related to psychological distress, whereas VN was directly related to psychological distress. Since VN is a residual factor in our study, the result means that it is not the global measure of GN that is related to psychological distress, but only the VN that exceeds the measure of GN. Individuals who are less vulnerably narcissistic than would be expected based on their GN report lower psychological distress. These findings corroborate existing research on the role of coping flexibility and emotion regulation in GN and VN. Ng et al. (83) identified more flexible coping with stress in GN as a crucial mediating factor that makes them appear psychologically healthier than VN. Di Pierro et al. (53) emphasized these differences in accessing adaptive emotion regulation strategies by demonstrating that VN was associated with emotion regulation difficulties, and in understanding, accepting, and being clear about emotional states, whereas GN was not. Also, Zhang et al. (84) showed that VN was positively correlated with emotion dysregulation. Fernie et al. (62) found that unlike GN, VN was significantly associated with the use of denial as coping with stress response when controlling for anxiety and social desirability and behavioral disengagement. Hansen-Brown and Freis (63) found that a hostile attribution bias is exclusively found in VN, not in GN.

Intriguingly, the mediator analysis of defense mechanisms on the relationship between GN and VN and psychological distress seems to turn the tables for GN: Even though not directly related to psychological distress, GN showed significant positive associations with psychological distress when mediated by maladaptive defense mechanisms. These findings strongly highlight the central role of defense mechanisms in understanding the concept and pathological core of grandiose narcissism. The underlying defensive structure of the grandiose facet seems to expose its vulnerability and furthermore explains the relationship between GN and VN. A study of Mielimaka et al. (61) found similar results for narcissism, which can be considered comparable since the used measures base their operationalization of narcissism on its grandiose facet: they found that pathological narcissism itself was not directly related with interpersonal problems but indirectly related when mediated by neurotic defense mechanisms. Following this thought, an interesting finding of Jauk and Kaufman (40) on the relationship between GN and VN revealed that solely the severity of grandiosity explains the difference between the two facets and that GN and VN may be dissociable at lower levels of grandiosity but merge into an antagonistic core with signs of psychological maladjustment at higher levels.

### The Role of Defense Mechanisms in Diagnosing Personality Impairment

Our findings entail numerous clinical implications which are of particular relevance in the light of the current revisions of the DSM-5 classification and diagnostic approach toward personality disorders [for a review: (85)]. With the inclusion of the Level of Personality Functioning Scale [LPFS; (86)] in the appendix of DSM-5 Alternative Model for Personality Disorders (AMPD), a dimensional approach for diagnosing personality disorders on the dimensions identity, self-direction, empathy, and intimacy is introduced. The model suggests to abandon the prevailing categories and focus on diagnosing underlying impairments in personality functioning. To date, numerous studies have supported this dimensional model to be more accurate and clinically more useful than the categorical approach (87, 88). Even though defense mechanisms are not part of the LPFS, preliminary studies indicate that defenses may add to the assessment of severity of personality impairment (89). In a subsequent clinical analysis of outliers, whose markers for clinical severity were significantly underestimated by the rating of the LPFS, Kampe (90) found that these were mainly personalities with high expressions of GN. In a comparison with a measure that includes defense mechanisms (91) personality impairments were accurately detected with a measure assessing defense mechanisms. These preliminary findings in clinical case analyses support our findings on maladaptive defense mechanisms in GN as a core element of its potential psychopathology. It emphasizes that considering defense mechanisms into revisions of the model for diagnosing personality disorders would be helpful. Defense mechanisms had already been included in DSM-IV-TR but been waived again in further revisions (12).

### Theoretical Implications and Clinical Relevance

The phenomenon of narcissism is of particular interest to the clinical field. Due to numerous difficulties in diagnostic approaches of pathlogical narcissism, the improvement in psychotherapeutic treatment is frequently overestimated. Not rarely treatment courses of narcissistic patients end with unpleasant surprises and sudden dropouts, fights, or an inability to end the treatment and separate from the therapist (8). Overall, our findings extend recent studies showing the interpersonal burden pathological narcissism places on relationships, both in daily life (92) and in clinical settings (93). These challenging interpersonal patterns are visible in treatment complications like drop-outs (4), the need for tact and sensitivity and therapist's adaptiveness when dealing with problematic relationship patterns (94), underlying shame (95) and the need for the therapist to turn to both fragile vulnerable aspects and provocative grandiose aspects of pathological narcissism (96).

Often there is little change in the personality and a sense of guardedness in the patient. This struggle has led Kernberg to write an article on “the almost untreatable narcissistic patient” (7), explaining these difficulties and emphasizing the role of defense mechanisms: Due to the rigid defensive structure, these patients don't admit their mental difficulties and make a big effort to constantly impress the therapist with good behaviors, charming attitudes, and superficial improvement. These psychoanalytic conclusions have already been revealed in empirical studies: Dickinson and Pincus (97) found that GN and VN reported domineering and vindictive interpersonal problems but GN denied interpersonal distress whereas VN reported high distress. Kaufman et al. (42) demonstrated that GN was not correlated with psychopathology and positively associated with life satisfaction but was also associated with multiple indicators of inauthenticity. Arikan (59) indicated that narcissistic defenses strongly relate to a tendency to devaluate and stigmatize mental illness, whereas adaptive defenses do not. This might explain the guardedness in narcissism when it comes to admitting mental distress. Our conclusions on the central role of defense mechanisms in narcissism complement these findings and further contribute to possible explanations of clinical difficulties with important implications for therapeutic approaches. It strengthens the assumption of defense mechanisms being the heart of the narcissistic pathology, or as Kernberg termed it, narcissism itself being a “character defense” (17). Our findings show the two-sidedness of the narcissistic defense structure: on the one hand, it prevents the experience of psychological distress, and on the other hand it depicts the core of the pathology. The inclusion of defensive operations in the understanding, diagnostic and treatment of (grandiose) narcissism is hence important for an accurate and successful treatment (7). One treatment for (narcissistic) personality disorders especially developed based on this assumption is Transference Focused Psychotherapy (TFP) by the group of Kernberg (6, 98–100). Based on working with countertransference, this treatment focuses on the extraction, clarification, and interpretation of projective defensive operations, aiming to help the patients understand their mental representations and personality difficulties. Studies have supported the idea that besides symptom reduction, TFP successfully facilitates change from disorganized attachment representations to organized ones and leads to a notable improvement in mentalization abilities (99–106).

### On the Relationship Between Grandiose and Vulnerable Narcissism

Eventually, this study adds to the discussion of the global operationalization of the concept of narcissism with GN and VN as potentially underlying factors. In our non-clinical sample, we did not find GN and VN as distinct factors with our initial modeling attempts. Instead, we built GN as a reference factor and VN as a residual factor. This raises the question of the relationship between GN and VN and whether they are two sides of a medal or fluctuating, if not overlapping constructs. Understanding the grandiose side as a defensive shield to protect the self from the conscious experience of the vulnerable side, our findings offer another perspective on the relationship between GN and VN. This leads to the assumption that individuals with narcissistic features might as well oscillate between grandiosity and vulnerability, which is compatible with the implications of Jauk and Kaufman (40), Jauk et al. (41), Gore and Widiger (46), and Oltmanns and Widiger (47). Even though this would support psychoanalytic theory on the concept of narcissism, further empirical studies to explore this relationship are still needed.

### Limitations

Even though our findings are in line with expectations derived from psychoanalytic theory and supported by prior research in related fields, a limitation to our study is that our findings only are based on a non-clinical sample that might not represent the pathological expressions of the constructs sufficiently. Particularly, the current sample demonstrated relatively low levels of psychological distress. This might be especially relevant for the associations between GN and psychological distress which could be expected to be more strongly and directly related in a clinical sample with higher levels at the pathological end of the spectrum than in this relatively healthy population. Future studies should replicate the results with clinical samples with expectedly higher distress and pathological narcissism levels. Furthermore, it is important to note that even though psychoanalytic theory draws causal conclusions, our study represents correlations, and no causal implications can be drawn due the cross-section design of the study.

### Perspectives

Our study highlights the importance of the concept of defense mechanisms for the conceptualization, diagnosis, and treatment of narcissism. For further research and for possible further changes in the diagnostic dimensions of personality pathology, we recommend considering defense mechanisms as a relevant domain in narcissism and personality in general. Studies that strengthen this matter are still needed. As our findings only refer to a non-clinical sample, we furthermore recommend including the pathological spectrum of narcissism into further conclusions on the central role of defense mechanism. We believe that a deeper understanding of defense mechanisms in narcissism, personality pathology, and mental disorders in general would be useful for both research and clinical practice. Even though deriving from psychoanalytic theory, we emphasize the relevance of the concept of defense mechanisms for all traditions and approaches.

Overall, future research should not only assess the phenomenological manifestations of disorders in terms of symptoms but to also take underlying, shared mechanisms into account. As most psychological disorders are related to dysfunctional emotion regulation (107), the inclusion of more hidden, that is implicit and unconscious, ways of dealing with affects and stressors, may be a fruitful endeavor (108). This is also in line with research showing that most processes operate implicitly rather than explicitly (109, 110).

## Data Availability Statement

The raw data supporting the conclusions of this article will be made available by the authors, without undue reservation.

## Ethics Statement

The studies involving human participants were reviewed and approved by Ethikkommission der Psychologischen Hochschule Berlin Prof. Dr. T. Storck Am Köllnischen Park 2 10179 Berlin. The patients/participants provided their written informed consent to participate in this study.

## Author Contributions

LK conceptualized the study and drafted the manuscript. LK and CR coordinated the assessment. JB conducted the analysis. SH-S contributed to planning and implementation of the study and writing. All authors critically revised the manuscript, contributed equally, and approved the final version to be published.

## Conflict of Interest

The authors declare that the research was conducted in the absence of any commercial or financial relationships that could be construed as a potential conflict of interest.

## Appendix

**Table A1:** Model Fit Indices of all 20 structural equation models.

**Defense mechanism**	***χ^2^***	***df***	***p***	**RMSEA**	**CFI**
**Adaptive**					
Suppression	11.334	11	0.4157	0.011	1.00
Anticipation	15.509	11	0.1604	0.040	0.997
Humor	14.216	11	0.2213	0.034	0.998
Sublimation	20.094	11	0.0441	0.057	0.994
Rationalization	12.398	11	0.3345	0.022	0.999
Denial	16.110	11	0.1371	0.043	0.996
Dissociation	14.606	11	0.2013	0.036	0.997
**Intermediate/neurotic**					
Pseudo-altruism	10.771	11	0.4627	0.000	1.00
Undoing	16.622	11	0.1196	0.045	0.996
Reaction formation	13.113	11	0.2860	0.028	0.999
Acting out	22.354	11	0.0218	0.064	0.992
**Maladaptive**					
Splitting	15.660	11	0.1542	0.041	0.997
Autistic fantasy	18.743	11	0.0659	0.053	0.995
Projection	34.480	11	0.0003	0.092	0.984
Passive aggression	22.378	11	0.0216	0.064	0.992
Idealization	15.998	11	0.1412	0.042	0.997
Somatization	13.209	11	0.2799	0.028	0.999
Isolation	14.979	11	0.1835	0.038	0.997
Displacement	11.996	11	0.3639	0.019	0.999
Devaluation	19.049	11	0.0602	0.054	0.994

*The 20 models differ in the defense mechanism*.

*RMSEA = Root Mean Square Error of Approximation; CFI = Comparative Fit Index*.
